# Association of Familial Adenomatous Polyposis With Classic Papillary Thyroid Carcinoma and Acromegaly: A Case Report

**DOI:** 10.1016/j.aed.2025.08.012

**Published:** 2025-08-28

**Authors:** Santiago Orozco Montoya, Guillermo Salazar Villa, Alejandro Román González, Johnayro Gutiérrez Restrepo

**Affiliations:** 1 Department of Endocrinology, Faculty of Medicine, University of Antioquia, Medellín, Colombia; 2 San Juan de Dios Hospital, Rionegro, Colombia; 3 Somer Clinic, Rionegro, Colombia

**Keywords:** acromegaly, adenomatous polyposis, classic papillary thyroid carcinoma

## Abstract

**Background/Objective:**

Familial adenomatous polyposis (FAP) is caused by pathogenic variants in the APC gene and is typically associated with colorectal polyps and an increased risk of colorectal and other cancers.

**Case Report:**

This report presents a 40-year-old female patient with a history of FAP and classic papillary thyroid carcinoma who, during clinical evaluation and follow-up, developed signs and symptoms of acromegaly later confirmed by biochemical tests.

**Discussion:**

The relationship between acromegaly and colonic polyps, as well as with papillary thyroid carcinoma, depends primarily on insulin-like growth factor 1; the cribriform-morular subtype of thyroid carcinoma is linked to FAP through different molecular mechanisms, and its association with acromegaly had not been described, thereby opening the possibility of investigating the mechanisms underlying this connection.

**Conclusion:**

This case illustrates a unique association among 3 uncommon pathologies that had not been previously reported.

**Highlights**

• Acromegaly may increase the likelihood of developing certain types of malignancies, such as colon carcinoma or the folicular variant of papillary thyroid carcinoma
• Acromegaly, thyroid nodules, and colon polyps may be related through the effect of insulin-like growth factor 1, whereas cases of familial adenomatous polyposis (FAP) are associated with colon polyps and thyroid carcinoma, but not with acromegaly
• The cribriform-morular variant of papillary thyroid carcinoma presents differently in patients with FAP, as, although these tumors are of similar size to the sporadic form, recurrence is complete if total thyroidectomy is not performed

**Clinical Relevance**

We report the first described case linking familial adenomatous polyposis, papillary thyroid carcinoma, and acromegaly. The connection’s mechanisms are uncertain and merit further study. Clinicians should remain alert for additional endocrine manifestations in patients with familial adenomatous polyposis.

## Introduction

Familial adenomatous polyposis (FAP) is an autosomal dominant disease characterized by the development of multiple colonic polyps and an almost 100% lifetime risk of colon cancer.^1^, ^2^, ^3^ With an incidence of 1 case per 10 000 people and caused by a mutation in the APC gene,^3^, ^4^, ^5^ FAP may be associated with extraintestinal manifestations—usually benign—but in up to 1.2% of cases, it can be linked to well-differentiated nonmedullary thyroid carcinoma of the classic and cribriform-morular papillary subtypes, as first described in 1968 and 1999, respectively.^5^^,^^6^

Acromegaly is a rare disease, with an incidence ranging from 2.8 to 14 cases per 100 000 inhabitants,^7^ which can independently be associated with colonic polyps or thyroid carcinoma.^8^ However, there are no previous records of the coexistence of all 3 conditions in the same patient, as detailed in the clinical case presented below.

## Case Report

This case features a 40-year-old female patient who had been under follow-up since 2022 due to a history of classic papillary thyroid carcinoma and familial adenomatous polyposis. In 2019, she underwent total proctocolectomy and ileostomy, during which multiple villous adenomas with high-grade dysplasia were found. Subsequently, she underwent resection of a desmoid tumor on the abdominal and pelvic wall in 2021. In that same year, she was diagnosed with classic papillary thyroid carcinoma, necessitating total thyroidectomy with a classification of pT2N0Mx, stage I, and low risk. To date, she has shown an excellent response.

The patient has a family history in which 3 sisters experienced the same gastrointestinal condition, and her mother had colon cancer. Genetic testing confirmed a pathogenic variant in the APC gene: Next-generation sequencing MyRisk Hereditary Cancer identified a heterozygous APC c.3927_3931del (p.Glu1309Aspfs∗4) mutation in 2021. No other relevant antecedents were noted. She also underwent ovarian endometrioma resection in 2023 and a subsequent resection of an abdominal wall desmoid tumor (left rectus abdominis) in April 2024. In the most recent imaging follow-up, multiple hepatic hemangiomas up to 28 mm in diameter were observed, along with a vesicular polyp (being monitored by ultrasound) and a stable pelvic mass suggestive of a desmoid tumor.

**Fig. 1:**
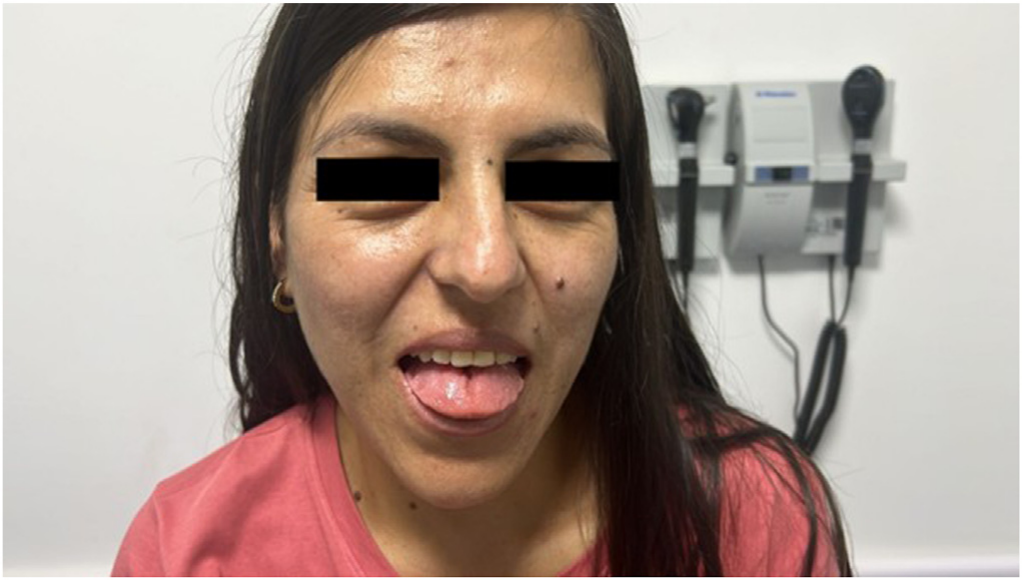
Patient’s appearance during follow-up: The patient has a slight prognathia and no macroglossia.

**Fig. 2:**
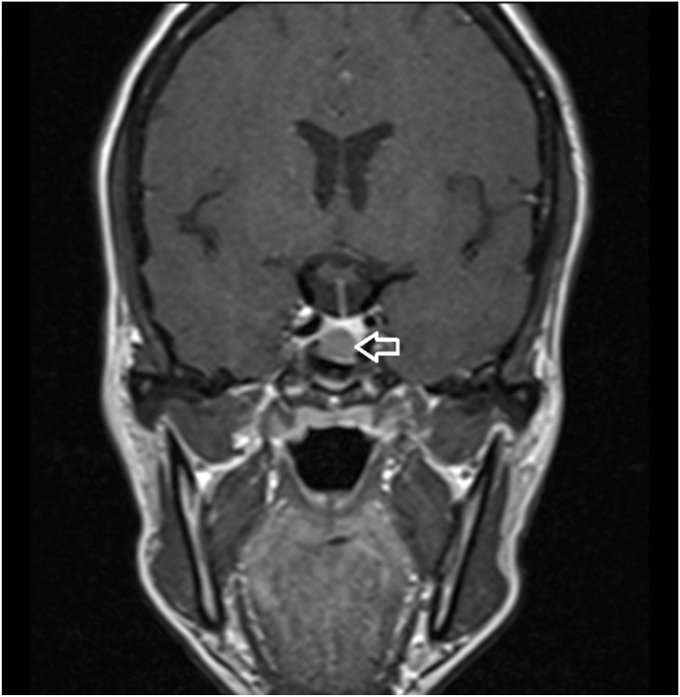
MRI of the sella turcica: Coronal T1-weighted image; the arrow points to the lesion in the adenohypophysis measuring 8.9 × 8.8 mm; slight deviation of the infundibulum; no invasion detected. *MRI* = magnetic resonance imaging.

During follow-up, she reported an increase in shoe size, profuse diaphoresis, headache, and menorrhagia, necessitating the initiation of continuous progestogen therapy to control bleeding. On physical examination, facial changes were noted, characterized by mild prognathism without macroglossia or dental diastasis. There was no acral enlargement (Fig. 1). Given the clinical suspicion, an insulin-like growth factor 1 (somatomedin C) measurement was ordered and performed in August 2024, yielding a value of 599.4 ng/mL (reference range 9–227 ng/mL), 2.6 times the upper limit of normal. A diagnosis of acromegaly was highly suspected, and additional studies (Table 1) and a pituitary magnetic resonance imaging were requested. In November 2024, a growth hormone suppression test was conducted and confirmed the diagnosis of acromegaly (Table 1), and a lesion measuring 8.9 × 8.8 mm was identified in the adenohypophysis, with slight rightward deviation of the infundibulum, without extrasellar invasion or optic chiasm involvement (Fig. 2). No metabolic disturbances or findings suggestive of sleep apnea syndrome were documented. Finally, she was referred to a pituitary neurosurgeon for surgical treatment, which was performed in February 2025; the pathological report of the surgical specimen was compatible with somatotroph adenoma (Table 2). The patient was reassessed in April 2025, with postoperative follow-up studies (Table 1).

**Table 1:** Hormonal Studies

Laboratory	Presurgical (November 18, 2024)	Postsurgical (February 27, 2025)
GH (0–120 min)	Baseline: 10.6; 30 min: 10.6; 60 min: 10.4; 90 min: 8.83; 120 min: 9.29 ng/mL (up to 8 ng/mL)	3.26 ng/mL (up to 8 ng/mL)
IGF-1	609.8 ng/mL (RR 69–227 ng/mL)	255 ng/dL (RR 83.6–223 ng/dL)
Cortisol (a.m.)	9.63 μg/dL	11.4 μg/dL
TSH	1.48 μU/mL	—
ACTH	26.2 pg/mL	15.5 pg/mL
Glucose	Baseline: 94 mg/dL; 2 h postload: 79 mg/dL	—
Prolactin	41 ng/mL	21.5 ng/mL

Abbreviations: ACTH = adrenocorticotropic hormone; GH = growth hormone; IGF-1 = insulin-like growth factor 1; RR, reference range; TSH = thyroid stimulant hormone.

**Table 2:** Histopathological and Immunohistochemical Findings

Location	Results	Comment
Midline tumor with sellar epicenter	Reticulum: Demonstrates loss of the normal pituitary acinar pattern. The histological findings, along with the immunohistochemistry profile, are suggestive of a growth hormone (somatotroph)-producing pituitary adenoma. PAS: Negative. Synaptophysin: Diffusely positive in neoplastic cells. p53: Nuclear positive in a moderate proportion of cells (interpreted as nonmutated). Ki67: Low proliferation index, less than 1%. CK cocktail: Negative (adequate external control). Growth hormone: Diffusely positive in the cells of interest. FSH: Focally positive (adequate external control). Prolactin, ACTH, TSH, FSH, and LH: Negative in the cells of interest. PIT1: Strongly and diffusely positive nuclear staining in neoplastic cells.	The histological findings combined with the immunohistochemical profile are suggestive of a growth hormone-producing pituitary adenoma (somatotroph adenoma).

Abbreviations: ACTH = adrenocorticotropi hormone; FSH = follicle stilulant hormone; LH = lutenizing hormone; PAS = periodic acid schiff; PIT1 = pituitary-specific positive transcription factor 1; TSH = thyroid stimulant hormone.

## Discussion

In this clinical case, we report what is, to date, the first known instance of several uncommon pathologies coexisting in the same patient—namely, FAP associated with classic papillary thyroid carcinoma and acromegaly secondary to a pituitary microadenoma. Independent associations between acromegaly and the development of malignancies have been described, attributed to the mitogenic effects of growth hormone and insulin-like growth factor 1,^8^ but never in relation to a genetic syndrome such as FAP. Multiple extraintestinal manifestations of FAP have been described, including hyperpigmented retinal epithelial lesions, osteomas, dermoid cysts, and dental abnormalities; moreover, this syndrome has been associated with malignancies of the thyroid, adrenal glands, and brain.^3^^,^^5^^,^^6^^,^^9^ In the context of FAP, the cribriform-morular variant (CMV) of papillary thyroid carcinoma tends to present with multiple tumor foci, absence of nodules at diagnosis, no extrathyroid extension, and is often identified through screening after FAP confirmation. Unlike sporadic CMV, recurrence is more likely—particularly after hemithyroidectomy—supporting total thyroidectomy as the preferred approach.^10^

The correlation between acromegaly, colonic polyposis, and thyroid nodules is well documented, as is the association with colon and thyroid adenocarcinoma, mediated by physiological mechanisms different from those seen in FAP (which do not involve APC mutations) and not related to the follicular subtype of thyroid carcinoma or CMV.^7^^,^^11^, ^12^, ^13^ It is estimated that between 7% and 11% of patients with acromegaly develop thyroid carcinoma, primarily of the follicular subtype.^12^^,^^14^ Major case series have described an association between prolonged uncontrolled disease activity—typically over 12 to 15 months—and a higher incidence of thyroid carcinoma. Additionally, whereas the BRAF V600E mutation is found in up to 62.5% of tumor foci, in patients with acromegaly and papillary thyroid carcinoma, this mutation is present in less than 10%.^15^ With respect to tumor number, size, and extrathyroid extension, no significant differences have been noted between cases with or without associated acromegaly. Experimentally, a correlation between APC and acromegaly has been observed, as continuous growth hormone stimulation in animal models has been shown to lead to inactivation of tumor protein 53 and consequently APC, resulting in a loss of tumor suppression and increased cellular proliferation in the colon, culminating in neoplasia.^16^ It remains unknown whether elevated growth hormone in an individual with a mutated APC, as seen in FAP, has different prognostic implications in other tissues. The odds ratio for colonic polyposis in acromegaly is 3.6, and the odds ratio for colon cancer is 4.3^17^; additionally, colonic adenomas have been reported in up to 23.4% of patients with acromegaly.^18^^,^^19^ To date, only 1 case has been reported by Kato et al,^20^ which described the association between acromegaly, papillary thyroid carcinoma, and duodenal adenocarcinoma. In that case, the patient had a family history of thyroid carcinoma, and the diagnosis of acromegaly and duodenal adenocarcinoma occurred after the thyroid malignancy—clearly underscoring the potential association between intestinal neoplasms and acromegaly. Considering that the diagnostic delay in acromegaly may range from 5 to 14 years,^8^ and given the temporal sequence of diagnoses alongside biological plausibility, there may be a causal relationship between both acromegaly and FAP and the development of thyroid carcinoma, although we cannot rule out the possibility that this represents a chance occurrence.

In conclusion, we present a case involving the uncommon associations of FAP and classic papillary thyroid carcinoma with acromegaly. This represents the first case of its kind in the literature and highlights the known systemic associations of genetic syndromes while introducing a concurrent uncommon endocrine disorder with as yet unknown prognostic implications.

## Disclosure

The authors have no conflicts of interest to disclose.
